# A Multi-Site, Multi-Wavelength PPG Platform for Continuous Non-Invasive Health Monitoring in Hospital Settings

**DOI:** 10.1109/TBCAS.2023.3254453

**Published:** 2023-04-01

**Authors:** Stefan Karolcik, Damien K. Ming, Sophie Yacoub, Alison H. Holmes, Pantelis Georgiou

**Affiliations:** Department of Electrical and Electronic Engineering, Imperial College London, SW7 2AZ London, U.K.; Centre for Antimicrobial Optimisation, Imperial College London, W12 0NN London, U.K.; Centre for Antimicrobial Optimisation, Centre for Tropical Medicine, Ho Chi Minh City 700000, Vietnam; Centre for Antimicrobial Optimisation, Imperial College London, W12 0NN London, U.K.; Department of Electrical and Electronic Engineering, Imperial College London, SW7 2AZ London, U.K.

**Keywords:** Biomedical monitoring, optical sensors, photoplethysmography, pulse oximeter, wearable health monitoring systems

## Abstract

This article presents a novel PPG acquisition platform capable of synchronous multi-wavelength signal acquisition from two measurement locations with up to 4 independent wavelengths from each in parallel. The platform is fully configurable and operates at 1ksps, accommodating a wide variety of transmitters and detectors to serve as both a research tool for experimentation and a clinical tool for disease monitoring. The sensing probes presented in this work acquire 4 PPG channels from the wrist and 4 PPG channels from the fingertip, with wavelengths such that surrogates for pulse wave velocity and haematocrit can be extracted. For conventional PPG sensing, we have achieved the mean error of 4.08 ± 3.72 bpm for heart-rate and a mean error of 1.54 ± 1.04% for SpO_*2*_ measurement, with the latter lying within the FDA limits for commercial pulse oximeters. We have further evaluated over 700 individual peak-to-peak time differences between wrist and fingertip signals, achieving a normalized weighted average PWV of 5.80 ± 1.58 m/s, matching with values of PWV found for this age group in literature. Lastly, we introduced and computed a haematocrit ratio (*R*_*hct*_) between the deep IR and deep red wavelength from the fingertip sensor, finding a significant difference between male and female values (median of 1.9 and 2.93 respectively) pointing to devices sensitivity to Hct.

## I Introduction

SINCE its inception in the 1930’s [1], photoplethysmography (abbreviated as PPG) has been continuously applied in clinical and fitness health monitoring. With the progress in sensor design and digital systems, more complex PPG applications capable of performing cuff-less blood pressure estimation, respiration rate monitoring and blood component analysis became possible. This further combined with the established clinical measurements of heart-rate and oxygen saturation, makes PPG the subject of rising research interest. While still facing the omnipresence of motion artefacts and noisy datasets, the involvement of machine learning techniques in analysis of large databases of PPG waveforms show promising results [2], [3]. Additionally, PPG systems can be built using readily available off-the-shelf components, allowing mass-production and benefiting from economies of scale.

A clear need for these mass-deployable, wearable and non-invasive systems can be seen in regions affected by infectious disease outbreaks. Due to nature of these infectious diseases, there can be a significant strain on healthcare resources caused by rapid spread of infections and a surge in hospitalisations.

For example, dengue infects over 100 million people annually [4] in tropical and sub-tropical regions. Even with only 1–5% of the cases developing severe disease [5], it creates a huge burden for healthcare facilities. The current methods of patient triage and monitoring using routine vital signs are often limited by a lack of equipment and staff capacity in endemic settings which can lead to late detection of clinical deterioration especially in overcrowded wards during the peak infection season. The proposed system has the potential to alleviate the pressure on healthcare resources and nursing time by providing a low-cost, continuous physiological monitoring system, allowing early warning of patient deterioration. This paper presents a modular PPG acquisition platform that combines an array of wavelengths and two sensing sites to obtain non-invasive estimates of multiple physiological parameters. Due to its modular design, the sensory probes can be changed according to requirements of the patient. The platform has been designed to allow synchronised signal acquisition from multiple body locations in parallel. Individual sensory probes have been designed for sensing of physiological parameters routinely measured in infections like dengue as the first application of the system. The ambition of this work is to become a base for complex PPG model development and validation, beyond the usual pulse oximeter or heart-rate monitor.

The paper starts by introducing the basics of PPG sensing and theoretical background on vital sign estimation from PPG. It then presents the platform in technical details, both hardware and software. Lastly, the experimental section shows the current capabilities of the platform with discussion interpreting the results and outlining the next steps.

## II Sensing Theory

### A PPG

**Fig. 2:**
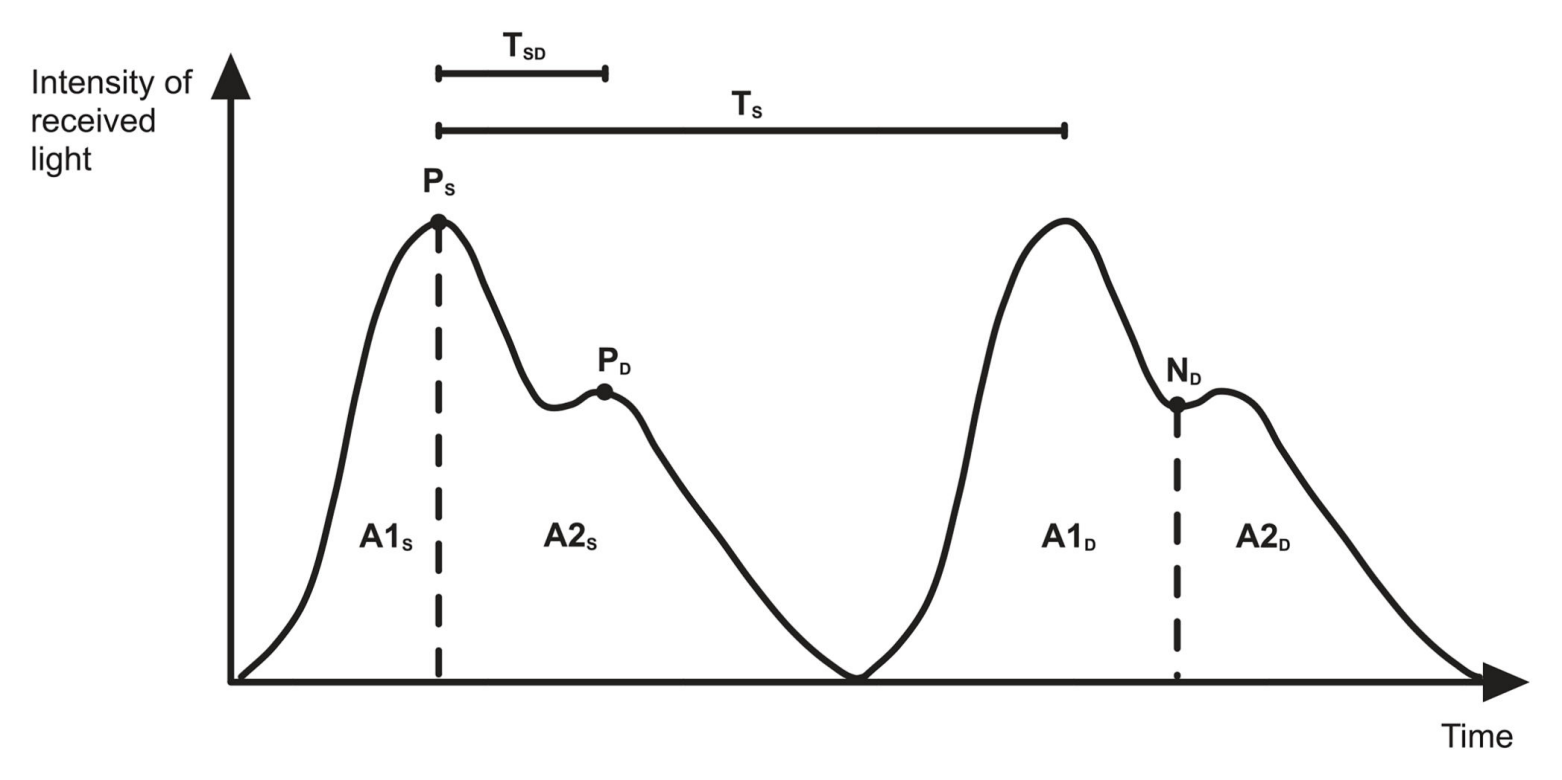
PPG waveform shape and most commonly extracted features. The figure contains 2 periods of the waveform with clearly defined dicrotic notch that is acquired from a fingertip sensor. The common features include: **T**_**SD**_: Time period between systolic and diastolic peak during a single beat, also related to large artery stiffness index [6]; **T**_**S**_: Time period between two consecutive systolic peaks, also corresponding to the heart rate and heart rate variability when measured over longer periods; **P**_**S**_: Systolic peak; **P**_**D**_: Diastolic peak; **N**_**D**_: Dicrotic notch, the slight increase in the pressure in the beginning of diastole caused by the closure of the aortic valve; **A1_S_** - **A2_S_**: Area under the curve split by the systolic peak; **A1**_**D**_ - **A2**_**D**_: Area under the curve split by the dicrotic notch.

Photoplethysmogram is obtained by illuminating skin using a light source at visible or near-IR wavelength and capturing the transmitted light with a photodiode. As light travels through the tissue, multiple absorption and scattering events occur, allowing to extract vital biological information on the receiver side. When sensing PPG, either on a fingertip or a wrist, most parts of the tissue are static and therefore their absorption can be approximated as constant provided the subject is at rest. The constant absorption then manifests as a DC offset in the resultant PPG waveform. The main contributor to the alternating part of the PPG waveform, pictured in Fig. 2, is the periodic blood flow caused by the cardiac cycle. Every time the heart pumps blood through the arteries and vascular network, the volume of blood at the sensed site momentarily changes, creating peaks and troughs in the waveform. This allows direct extraction of heart-rate provided the peaks are clearly distinguishable. It is important to note that PPG systems are notably susceptible to motion artefacts as the initial approximation of constant absorption of surrounding tissue is no longer valid when the subject is moving.

Conventional PPG systems are built with a particular application in mind, allowing optimization of the acquisition circuitry for either heart-rate or oxygen saturation measurement. The receiver pipeline consists of a photodiode connected to a trans-impedance amplifier, followed by a gain stage and an analogue-to-digital converter with optional analogue filtering on the way. In this work, we have leveraged the configurability provided by the analogue front-end chip developed by Texas Instruments, the AFE4900, to allow PPG waveforms capture at mutlitudes of wavelengths simultaneously. This design decision further allows construction of new sensory probes in the future with little constraints on the sensing circuitry components.

### B Light Absorption

PPG analysis can provide information on other physiological parameters due to the varying absorptivity of light wavelengths across the visible and IR spectrum with respect to blood constituents. This approach can be explained by the Beer-Lambert Law of Absorbance, applied to the blood vessels with the following equation [7]:

(1) A=ϵ(λ)lc

Where A is the absorbance, *ϵ* (*λ*) is the absorption coefficient of the component in the measure sample at specific wavelength (λ), *l* is the optical path length through the sample and *c* is the concentration of the component in the measured sample.

**Fig. 3:**
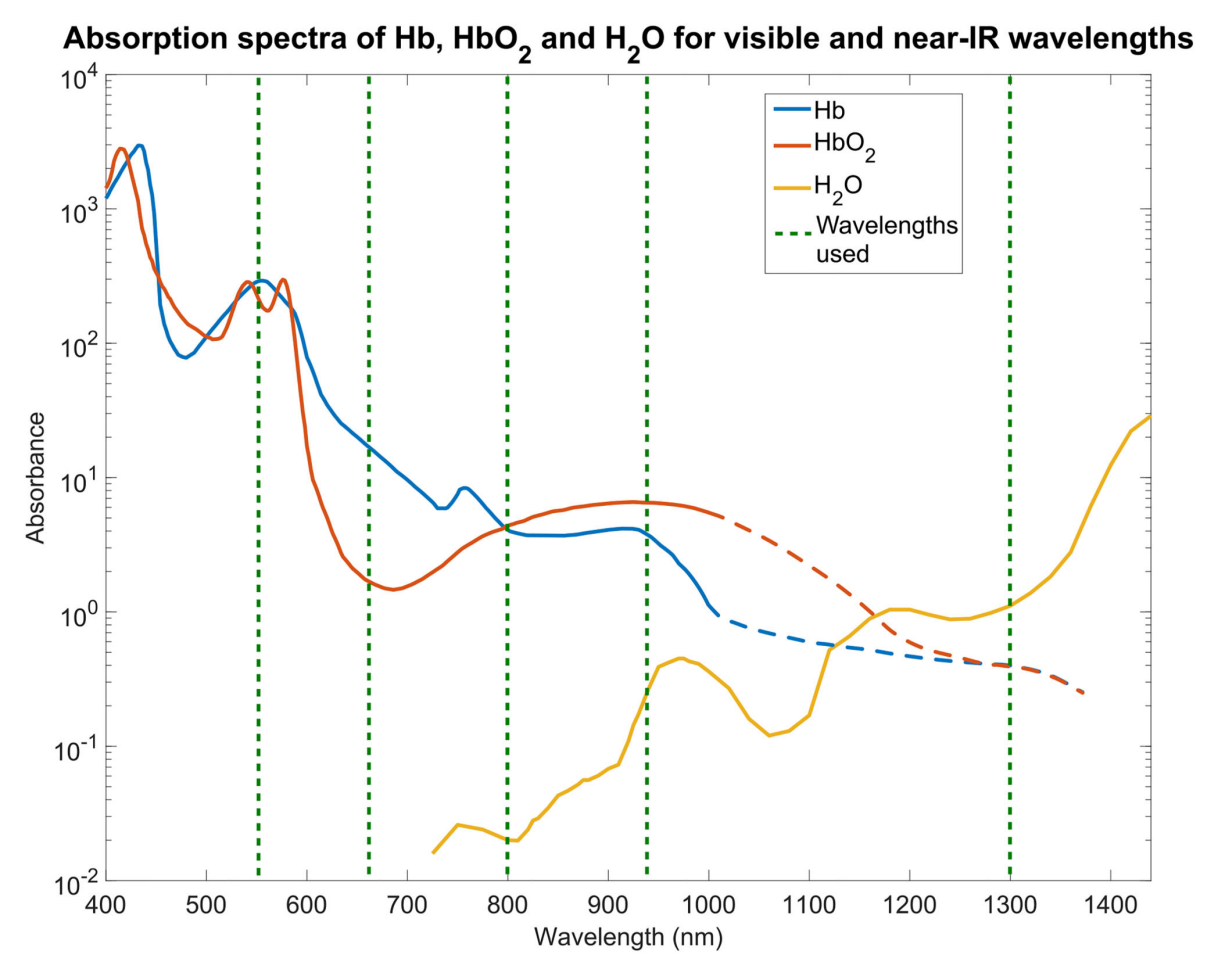
Light absorption properties for various blood components and water across the visible and near-IR light spectrum. The solid lines are based on spectroscopy data compiled by S. A. Prahl [8]. The dashed continuation of lines correspond to the approximate absorbances based on the work of Schmitt et al. [9] and Kuenstner et al. [10]. The green horizontal lines correspond to the wavelengths used within this work.

The Fig. 3 shows the varying absorbance of blood constituents at different wavelengths. Combining specific wavelengths in a single sensor probe at similar or identical optical path lengths, allows to cancel out parts of the Beer-Lambert equation and obtain the concentration estimate for given constituent.

### C Estimation of Physiological Parameters From PPG

Established commercial applications of photoplethysmography are limited to heart-rate monitoring using smartwatches and smartphones and fingertip pulse oximetry for oxygen saturations in clinical settings [11]. With the decreasing cost of PPG sensor components, miniaturisation of specialised analogue circuits and advancements in machine learning, new techniques for estimation of physiological parameters are being developed. These include estimation of blood pressure and haematocrit. The cuff-less blood pressure estimation in particular has been a focus of research for more than a decade [12], [13], [14] as solving this using an inexpensive and non-invasive sensor based on PPG would have a major impact on tackling hypertension, which causes significant morbidity and mortality globally [14]. The modern machine learning based approaches for estimating BP show promising results [15] but their mass adoption is hindered by inconsistencies between different patients and susceptibility to noise [16].

#### 1) Heart Rate (HR)

Looking back at Fig. 2, the heart-rate peaks are the most prominent features of the PPG waveform. Therefore the only parameter needed to calculate HR is sampling frequency. By not requiring to capture any more features except the peaks, most fitness applications choose green wavelength as it does not penetrate the tissue as deep as longer wavelengths and therefore is more resistant to motion artefacts at the cost of being less feature rich [14].

#### 2) Pulse Oximetry (SpO_2_)

In clinical settings, PPG is used to measure oxygen saturation (SpO_2_) of the blood also known as pulse oximetry. Oxygen saturation is calculated directly from ratios between PPG signal waveforms at two distinct wavelengths. The two values are selected to maximize the difference between absorption in oxygenated hemoglobin (red blood cells that carry oxygen) and de-oxygenated hemoglobin (red blood cells not carrying oxygen). The commonly selected wavelengths are 660 nm (red) and 940 nm (infrared). Looking at Fig. 3 a clear difference in absorption can be observed, with the red wavelength being dominated by Hb and the infrared being dominated by Hbo_2_. Using the approach described in [17] a ratio R is calculated using the formula below:

(2) $$R\approx \frac{AC\left(\lambda_{1}\right)/DC\left(\lambda_{1}\right)}{AC\left(\lambda_{2}\right)/DC\left(\lambda_{2}\right)}$$

Where *AC*(λ) is the pulsatile component of the PPG waveform at given wavelength *λ* and *DC*(*λ*) is the DC offset of the PPG waveform. This ratio R can be directly used to obtain an SpO_2_ value using a calibration curve [17].

#### 3) Haematocrit (Hct)

The theory behind Hct sensing is based on a similar principle to that of pulse oximetry. Haematocrit is defined as the proportion of red blood cells in whole blood. In the landmark paper published by Schmitt et al. [9], researchers argued that the rest of blood constituents (plasma) can be approximated to water in terms of light absorption. By employing this approximation, Hct can be estimated from the ratio between red blood cells and water. From Fig. 3, the two wavelengths picked in this work were 800 nm and 1300 nm. These values were intentionally chosen to be at the isosbestic point (the light absorption is the same for Hb and Hb0_2_) so that varying values of SpO_2_ will not interfere with the measurement. The main downfall of using this method is that at 1300 nm, the absorption of water is the dominant factor and water is present across the human body, not only in blood which highly attenuates the transmitted light intensity. Continuing the work of [9], new approaches used arrays of multiple wave-lengths and tested haematocrit detection in-vivo on 43 [18] and 549 [19], [20] people showing a correlation coefficient of over 0.68.

This work aimed to design a sensor that combines the multi-wavelength approach with the original landmark proposal for 2 isosbestic wavelengths by creating a 4-wavelength array.

#### 4) Blood Pressure (BP)

Estimating blood pressure from PPG has been attempted by a number of techniques with varying accuracy and results. The most accurate analytical approaches do not use the PPG signal alone but employ a wider variety of bio-signals, mainly ECG [21]. To estimate the blood pressure from a combination of PPG and ECG, a surrogate called Pulse transmit time (PTT) is extracted from the data. PTT is defined as the time difference between the peaks of PPG and ECG waveforms and it was shown to correlate well with blood pressure despite the requirements of frequent re-calibration [22]. Building a system around PTT brings additional design challenges making the system more complicated. Requiring electrical coupling to the patient for ECG electrodes and multiple contact points on the body makes the system uncomfortable to wear for long periods and impractical for non-hospitalised patients.

An alternative method in [12] used two PPG sensors spaced a known distance apart to calculate pulse wave velocity (PWV) and in turn convert to blood pressure using the equation:

(3) $$P=k_{1}\;ln\left(c^{2}\right)+k_{2}$$

where P is the blood pressure, c is the pulse wave velocity and *k*_1,2_ are constants that need to be obtained during calibration.

Thanks to the evolution of data science in healthcare, researchers have been attempting to estimate blood pressure from PPG waveform alone, using its rich, previously underutilised feature set. Looking back at the Fig. 2, the combination of some of the illustrated features have been successfully used to train neural networks for BP estimation from PPG [23], [24]. The research team in [23] managed to obtain results acceptable under the AAMI [25] strict limits with mean error of 3.80±3.46 mmHg for systolic and 2.21±2.09 mmHg for diastolic BP.

The presented system will start by extracting the PWV surrogate first before applying machine learning approaches in future work.

## III Platform Implementation

The platform implementation combines our understanding of PPG sensing capabilities with sensing of the main physiological parameters measured in patients with dengue. In the minority of cases that progress to severe disease this manifests as low blood pressure from vascular leak known as dengue shock syndrome. Increased capillary permeability resulting in plasma leaking out of vessels results in an increasing haematocrit and decreasing blood pressure [5]. As these parameters are usually constant in healthy subjects, dengue patients allow a unique opportunity to track deviations as opposed to providing absolute values of vital signs. This way we can relax the requirements for accuracy of vital sign estimation and instead focus on tracking changes throughout the course of the disease.

The prototype has been assembled using a specialised, off-the-shelf Texas Instrument chip range, the biosensing analogue front-end (AFE). Specifically, we used the AFE4900 equipped with 4 independent LED channels and up to 3 independent photodiode inputs. A range of configuration options allows each of the LED channels to be separately configured for variable gain, DC offset current and LED forward current. The platform can therefore support a large selection of off-the-shelf LED’s and photodiode pairs at varying wavelengths. All settings are stored in internal registers of the chip, configured via SPI. For the first iteration of the platform, a modified version of the development board provided by TI was used. Miniaturisation is another avenue provided by this particular chip range selection. The whole system has been carefully designed to not require any physically large components laying the foundation for an eventual wearable implementation. This allows the portable second generation of the system to utilize the same sensing approach and processing techniques as will be validated with the presented prototype.

To allow acquisition from multiple body sites, the prototype platform contains 2 of these development boards each connected to a custom made sensor probe. Each AFE4900EVM board is setup to use the internal oscillator at 1.024 MHz, leading to 1024 clock cycles per sample at 1 kHz sampling frequency.

Five different wavelengths across eight distinct PPG channels are acquired, with each sensing location having four channels. The four channels from wrist are acquired in parallel with the four channels from finger. The pulse oximetry wavelengths (660 nm and 940 nm) are present in both probes but due to high noise levels in the reflective wrist probe, only the ones acquired from transmissive fingertip probe are used in experiments. In total, the trasmissive finger probe contains 4 wavelengths: 660 nm, 800 nm, 940 nm and 1300 nm - corresponding to the wavelength for pulse oximetry and haematocrit sensing combined. The reflective wristwatch style probe contains 3 wavelengths and an ambient channel: 525 nm, 660 nm and 940 nm. Here, only the green channel PPG waveform is used to calculate pulse velocity across the hand and confirm heart-rate readings.

### A Probe Design and Location

Probes have been custom designed to accommodate for the required wavelengths and sensing locations. Different locations affect the shape and feature set of the PPG waveform and therefore care needs to be taken when selecting the correct one for the given application. Requirements for the locations were as follows: comfortable to wear for long periods of time, acceptable quality PPG signal available and close proximity of the two locations. Based on the PPG location evaluation in [14] fingertip and wrist were picked as optimal spots for measurement. As fingertip sensors are widely designed as clips that completely obscure the end of the finger and make the hand hard to use, an alternative, ring-like design is proposed taking inspiration from an existing pulse oximeter sensor design by Viatom (CheckMe O2).

#### 1) Wrist Probe

**Fig. 4:**
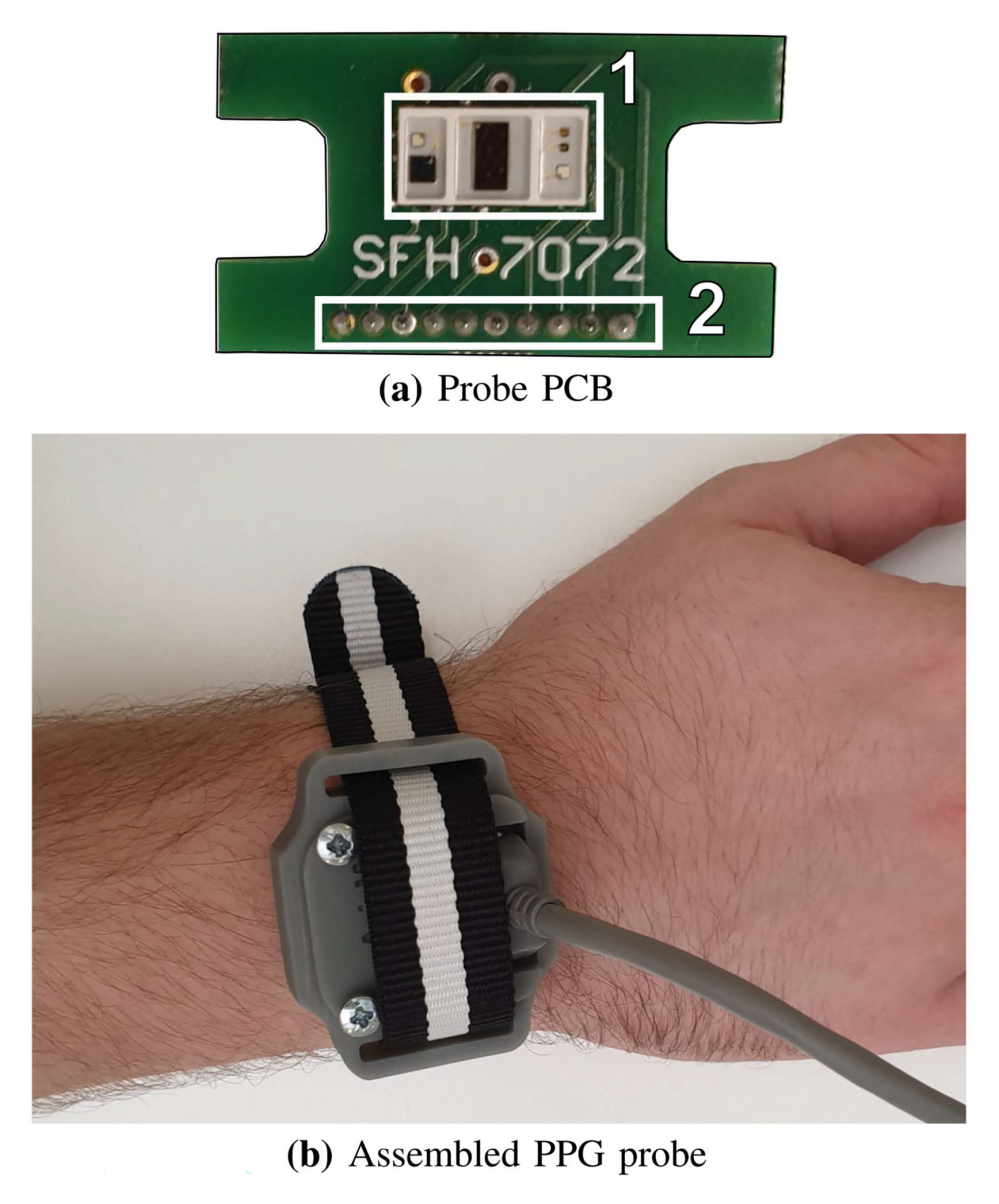
The wrist probe design inspired by classic watch, houses the PCB inside a 3D printed structure secured on hand by using a flexible strap. The 10-lead medical cable is directly connected to the probe. The PCB pictured in 4(a) contains the PPG array sensor SFH7072 from OSRAM (1) and 10-lead connector (2).

A watch-like design allows comfortable wear while providing good adhesion to the skin. The probe casing is custom 3D printed from Fig. 4 TOUGH-GRY 10 (3DSystems). The PCB inside contains an off-the-shelf reflectance PPG LED array SFH7072 (OSRAM) containing the 3 wavelengths: 525 nm, 660 nm and 940 nm with one broadband photodiode and one IR-cut photodiode with improved sensitivity to visible wavelengths. Standard wrist-watch style band allows accommodation of various wrist sizes. The inner PCB and fully assembled wrist probe are illustrated in Fig. 4.

#### 2) Finger Ring Probe

**Fig. 5:**
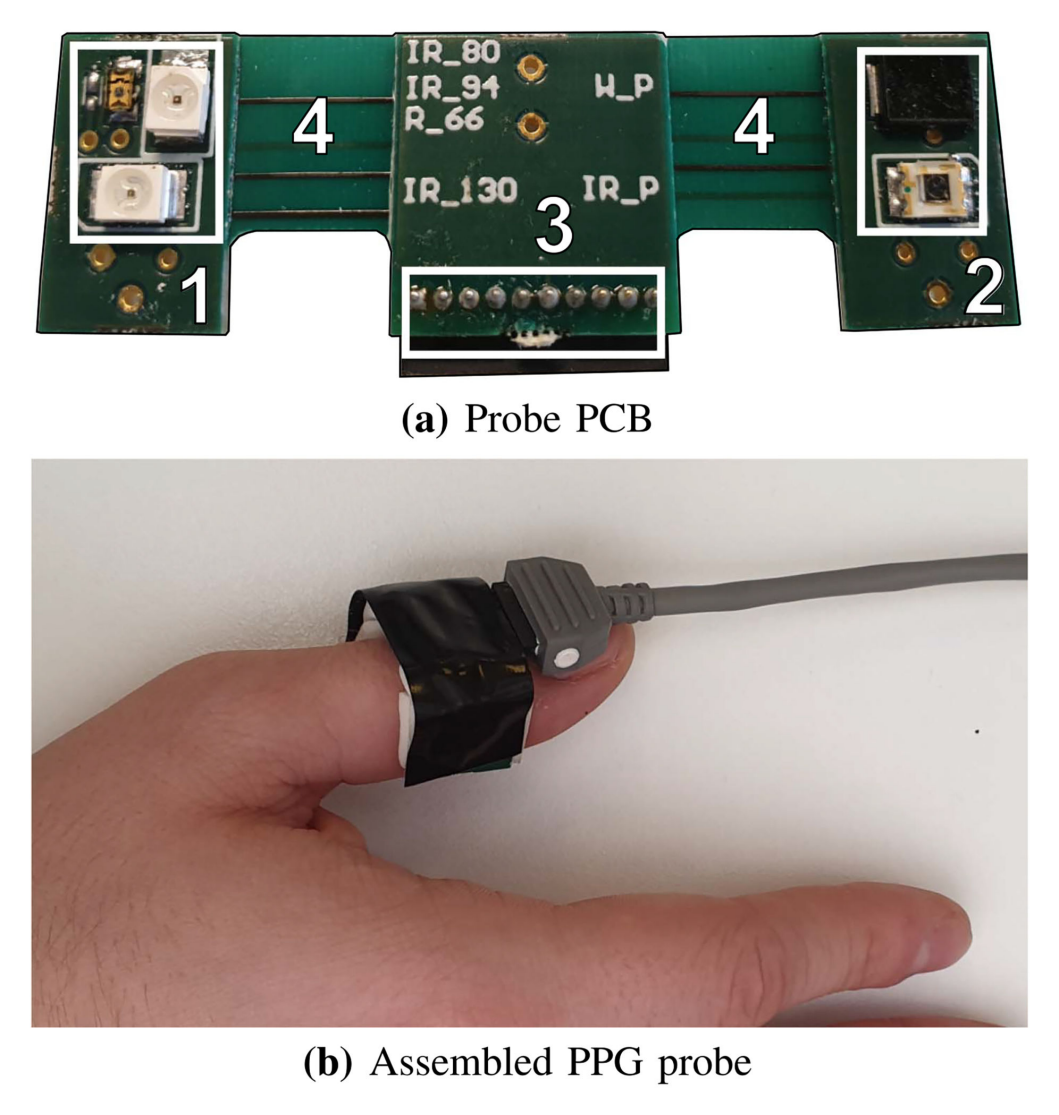
The semi-flex PCB design of the ring probe provides tight fit on variety of finger shapes and thicknesses. For best results, the sensor should be placed on the top part of the finger or directly on the fingertip. The pictured PCB design in 5(a) contains the emitter region (1), detector region (2), 10-lead connector (3) and flexible regions allowing 90^°^ bends (4).

The finger probe design utilizes the semi-flex PCB technology to accommodate various finger sizes. As pictured in Fig. 5 the board consists of 3 rigid parts and 2 flexible interconnects that allow the PCB to loop around the finger. Sensing array is constructed from discreet off-the-shelf components. Total of 4 medical grade SMT package LEDs are used on one side to provide the emitting part of the PPG sensor. There are two photo-diodes on the receiver side, the first one being a standard broadband photodiode sensitive to light wavelengths from 500 nm to 1000 nm. The second is an InGaAs photodiode sensitive in the 1300 nm wavelength region paired with the deep IR LED. The PCB is encompassed in a flexible, rubber-like 3D printed material to allow tight fit on the finger. The electronically sensitive parts of the PCB are sealed in non-conducting epoxy and covered by a translucent plastic film to prevent shorts caused by sweat or accidental liquid spillage.

### B Hardware Implementation

**Fig. 6:**
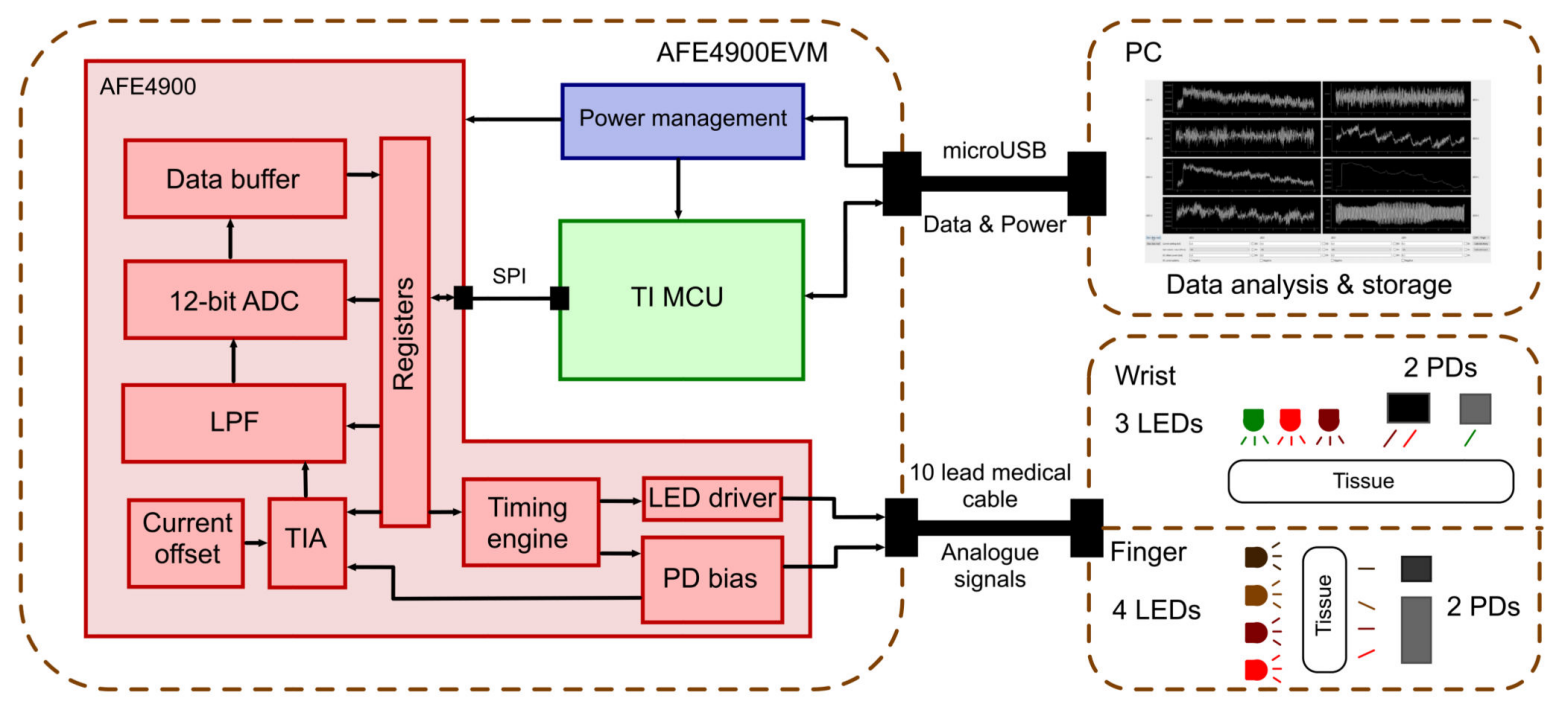
The block diagram of the presented PPG acquisition platform. The AFE4900EVM is an off-the-shelf development board from Texas Instruments, built around the AFE4900 analogue front-end chip for PPG. The chip (pictured in red) contains the whole pipeline for PPG signal with most of the block being configurable by internal registers. To configure this chip and retrieve data, an intermediary microcontroller (pictured in green) translates the standard serial commands from the PC. The probes utilise a 10-lead analogue medical cable with adequate shielding to prevent cross talk. The wrist probe contains 3 LEDs and 2 PDs in reflectance configuration, while the finger probe contains 4 LEDs and 2 PDs in transmissive configuration.

The block diagram in Fig. 6 illustrates the 3 main parts of the system. The analogue front-end chip is illustrated in red. This chip contains all necessary circuitry to acquire PPG waveform. On the emitter side, a configurable LED driver is used to drive up to 4 separate LEDs in parallel. The current going through each LED can be adjusted to values in the range of 0 to 200 mA. On the receiver side, the photodiode is fed directly into a transimpedance amplifier stage with an optional DC current offset which is then low-pass filtered and digitised. The chip is capable of having 3 different photodiode connected at the same time and multiplexes between them based on the timing controls. The timing registers inside of the AFE4900 guide the frequency of acquisition and define which LED should be paired with which photodiode. Each LED is only turned on for a fraction of the PPG acquisition period to preserve power and allow all 4 channels in a single cycle.

The internal register values were setup to acquire signal across a wide range of finger thicknesses. As broadband photodiode has varying sensitivity across the wavelength spectrum used, the TIA gain was adjusted accordingly. For 660 nm wavelength, TIA feedback resistor was set to 250 kΩ to counteract lower photodiode sensitivity while for 800 nm and 940 nm it was set to 50 kΩ. 1300 nm used a different photodiode and 25 kΩ feedback resistor was sufficient. Each LED was on for 95 µs, sampled for 70 µs and converted to digital value in additional 120 µs. The remaining 400 µs the AFE chip was put to sleep to preserve power. This was sequentially repeated 4 times for each AFE board in parallel to produce 8 PPG channels.

The acquired raw data for all 4 channels is digitised using a 12-bit ADC and stored in internal registers. The 12-bit values are extracted from the board via an embedded micro-controller implementing an USB serial port communication to PC. At the other end, the 10-lead medical cable connects to the sensor probe where the pins correspond to either an LED driver, photodiode biasing receiver or ground connection. The modularity of the design allows creation of new probes as long as the pin order of the connector is maintained and the sensing components are rated below the maximum ratings of the analogue chip. This way, any new probe design only requires a software configuration before it can be used for raw PPG acquisition.

**Fig. 7:**
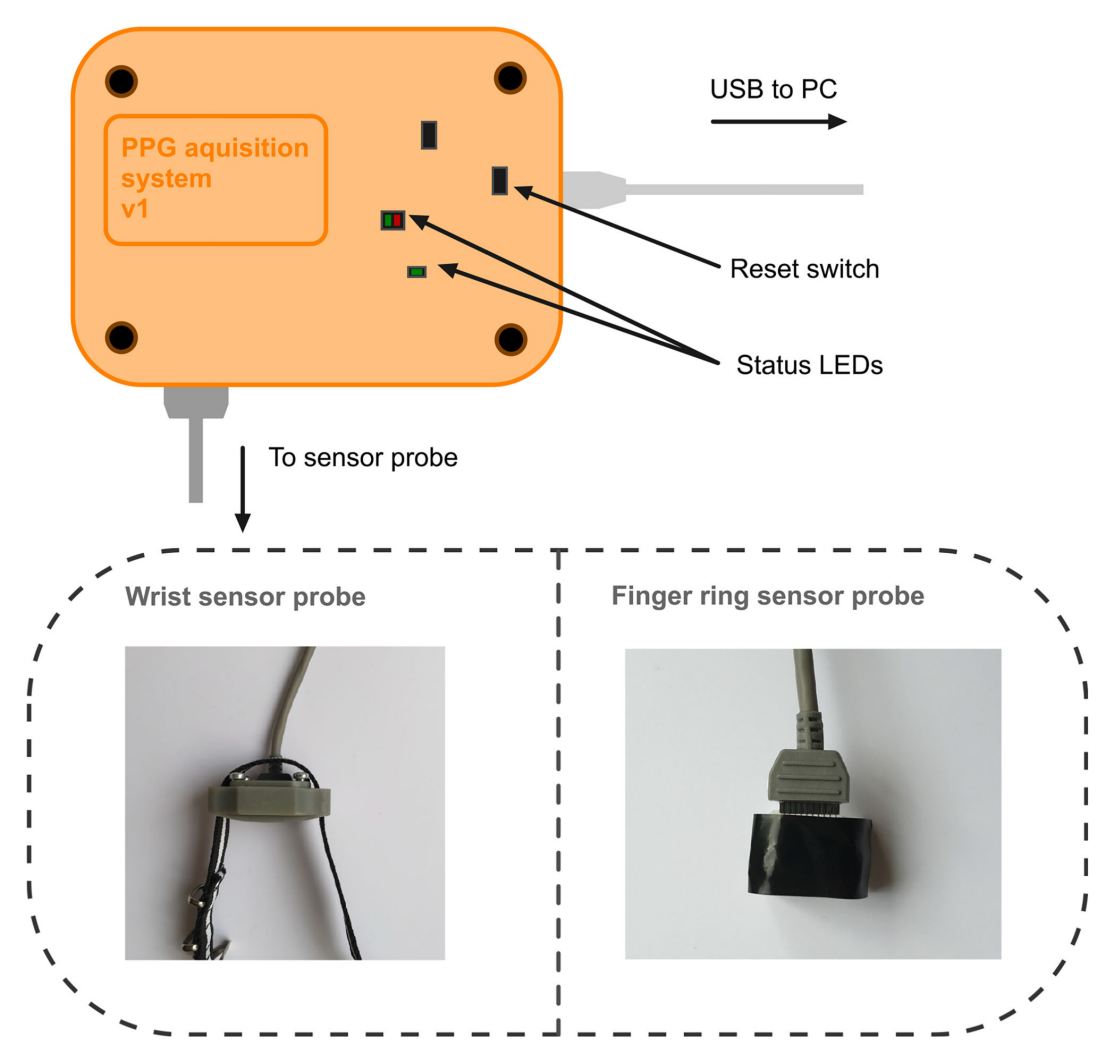
Illustration of all parts of the system. The tan-coloured 3D printed box contains the TI AFE4900EVM boards providing the analogue front-end for PPG acquisition and analogue-to-digital conversion of the acquired data. A custom designed probes with standardized 10-pin connector are connected on one side, while the microUSB cable is connected to PC from the other. 2 USB cables and 2 analogue cables are needed to operate two sensor probes in parallel.

The finished device is encased in a custom 3D printed case with 2 AFE4900EVM boards stacked on top of each other. The system is illustrated in Fig. 7. Each sensor probe is operating independently, connected to a separate TI board. The data is then synchronized in the accompanying software application after being received by the PC from two USB cables.

### C Software Implementation

One of the key elements of the proposed system is synchronized data acquisition with up to 8 separate PPG waveforms and raw data export for further analysis. To achieve this, a custom multithreaded Windows application was developed. The graphical user interface (GUI) was written in Python and visualizes all 8 possible LED channels with waveform data in real-time. In the backend, the system keeps reading both serial ports in parallel using separate software threads and assigns timestamps to datapoints as they come. Timestamps are saved together with the data to allow synchronisation between the two boards even if their internal clocks are not matched. Saving to file is incremental, protecting the system from complete data loss in case the system would stop responding for any reason. Communication between the board and PC is implemented via a proprietary TI serial protocol shared under an NDA and customized for parallel multi-board acquisition.

## Experimental Data Acquisition

**Fig. 8:**
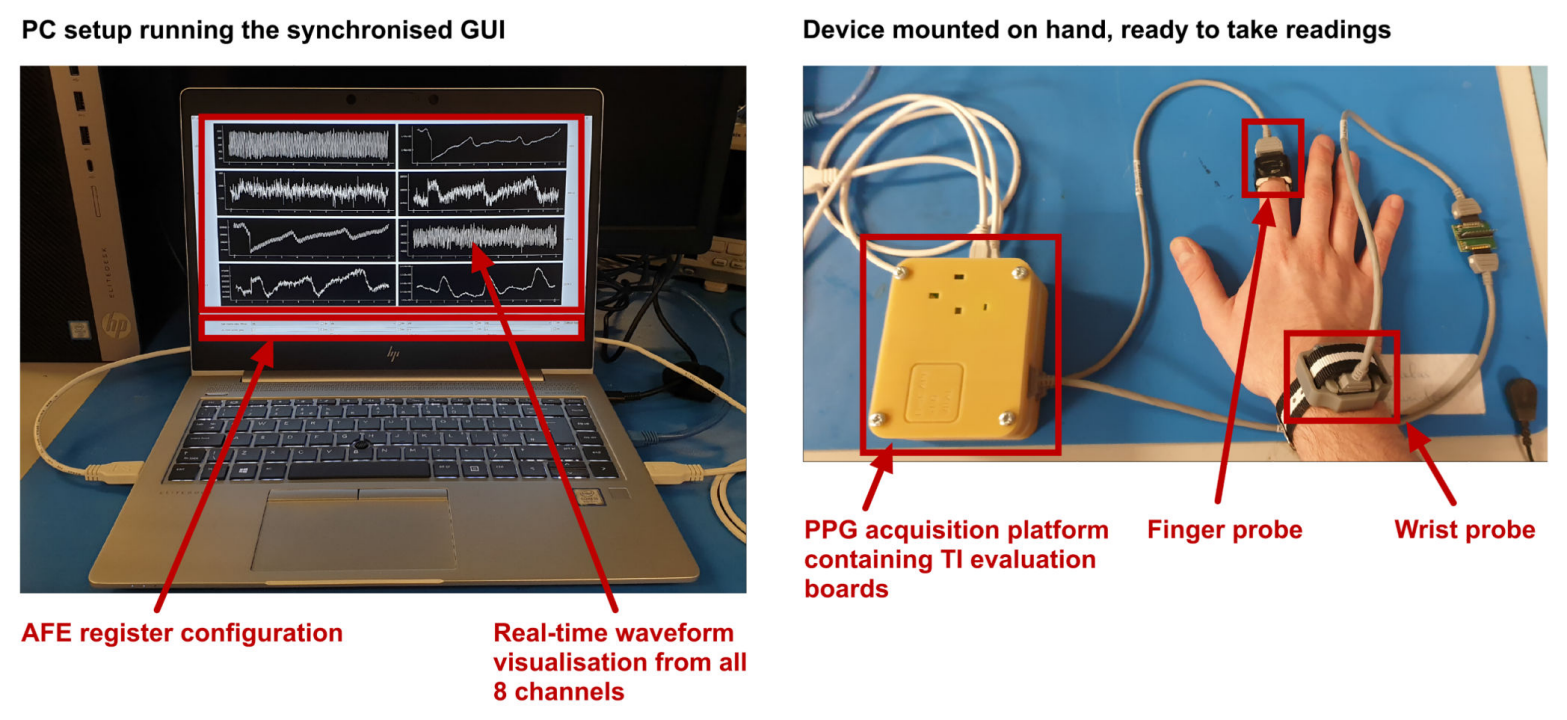
Diagram showing the experimental setup. Two sensors are mounted on the subject’s hand on finger and wrist respectively. The Windows application GUI running on the PC is showing real-time data stream coming from the device from all 8 wavelength channels. The visualised data is raw and unfiltered to not introduce additional strain on computing resources.

To verify the usability and accuracy of the proposed platform, a healthy volunteer study was conducted at Imperial College London. The study was approved by the University ethics committee (ICREC) reference 19IC5568, and in total involved 10 participants (4 female, 6 male) aged 23-32. Data were acquired from both sensor probes on one hand while a clinical-grade pulse oximeter (Masimo Sat901+) obtained measurements from the other hand. The Fig. 8 illustrates the experimental setup with both probes positioned on the subject’s right hand. A Windows laptop was used to visualise the data in real time and save them locally. To obtain a measure for pulse wave velocity, the highest supported sampling frequency of 1 kHz was chosen. When sampling at this frequency, the resultant file size for all 8 channels together is approximately 12 MB per 1 minute of recording. While significant, the modern SSDs are fast and large enough to support multi-hour continuous recordings.

### A Example Waveforms

**Fig. 9:**
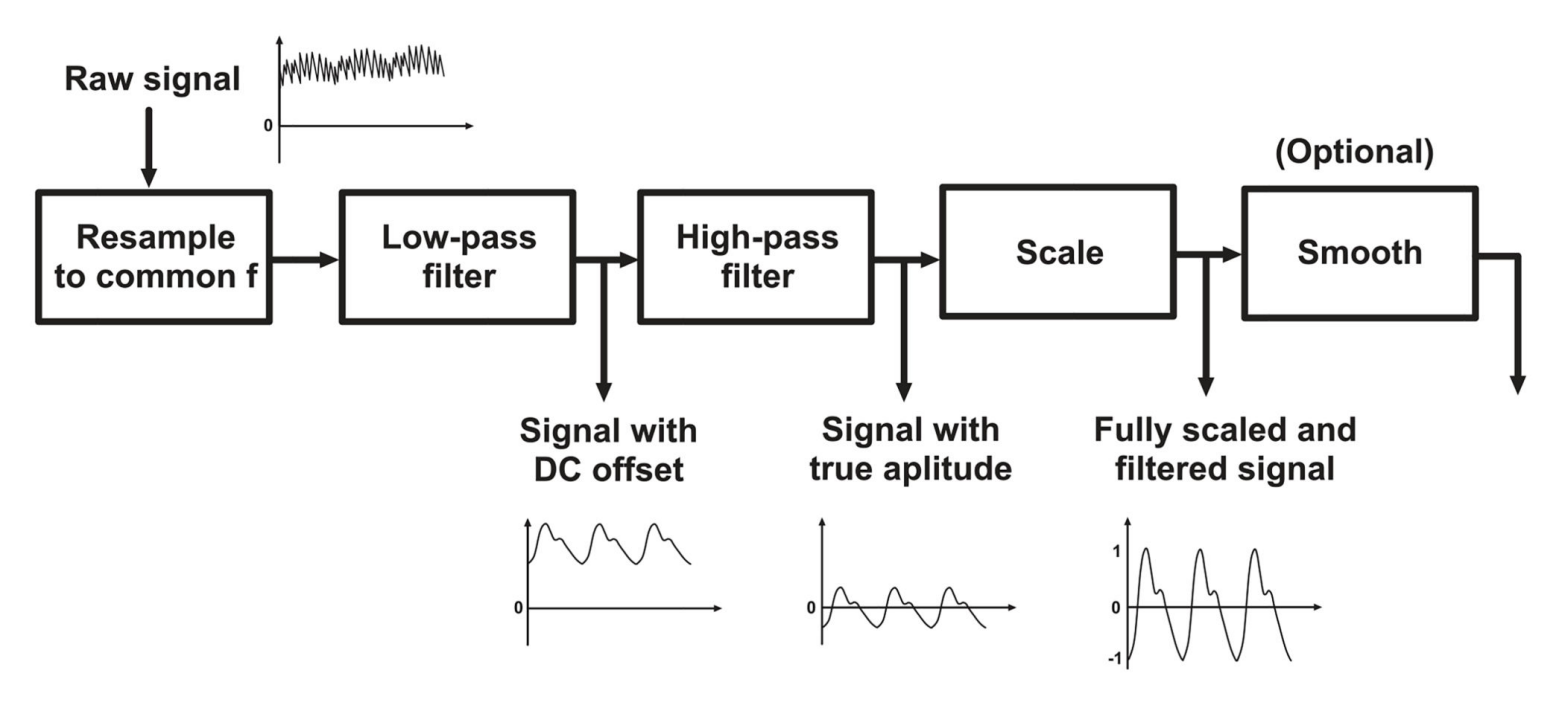
An illustration of the digital signal processing pipeline used on the raw data from obtained from the system. In the first stage, signals from the wrist probe are re-sampled to match the finger probe frequency and synchronised. The second stage employs a low pass filter with the cutoff frequency of 20 Hz that removes the high frequency noise including the 50 Hz mains interference. In the next stage, high-pass filter with a cutoff frequency of 0.5 Hz removes the DC offset. Lastly, the scaling step allows precise morphology-based feature extraction. This step is only applicable for features that do not depend on true amplitude. An optional step of smoothing is sometimes performed for low amplitude signals.

As the two sensor probes operate independently, data timestamps for synchronisation are processed before signal processing starts. In most cases, the AFE4900EVM boards internal oscillators are not matched causing difference in sampling rates of up to 12 Hz between the two boards when acquiring data at 1 kHz. To combat this, the true sampling rate is extracted from saved timestamps during post-processing and waveforms from one board are resampled to match frequency with the other. Using their respective starting timestamps the two boards are then synchronized together to a common starting point for all acquisition channels. The system was validated by a series of experiments, recording both sensor probes across all available wavelengths. Throughout the experimental section, the raw signal is analysed using an unified signal processing pipeline as illustrated in Fig. 9. The pipeline has 5 stages:

1) Resampling stage: Data from wrist probe are resampled to match frequency of finger probe and synchronized into a single large matrix.
2) Low-pass stage: Data is filtered with a Butterworth filter with cutoff frequency of 20 Hz. At this point an alternative snapshot of the dataset is saved, to be used for DC part of the AC/DC ratio calculation in pulse oximetry.
3) High-pass stage: Employing a high-pass Butterworth filter with 0.5 Hz cutoff to removes DC offset from the signal. Snapshot of the dataset is saved again, to be used for the AC part of the AC/DC ratio calculation in pulse oximetry.
4) Scaling stage: The signal is scaled between 1 and -1, pronouncing features and allowing consistent peak detection for heart-rate and PWV analysis.
5) (Optional) Smoothing stage: Employing Savinsky-Golay filter, this stage allows reconstruction of low amplitude PPG suffering from non-periodic noise.

The final pipeline output scales and normalizes the waveform to allow precise feature extraction and accurate peak detection. For SpO_2_ or other ration based calculations, the intermediate results of the pipeline are used instead.

#### 1) Finger Recordings

**Fig. 10:**
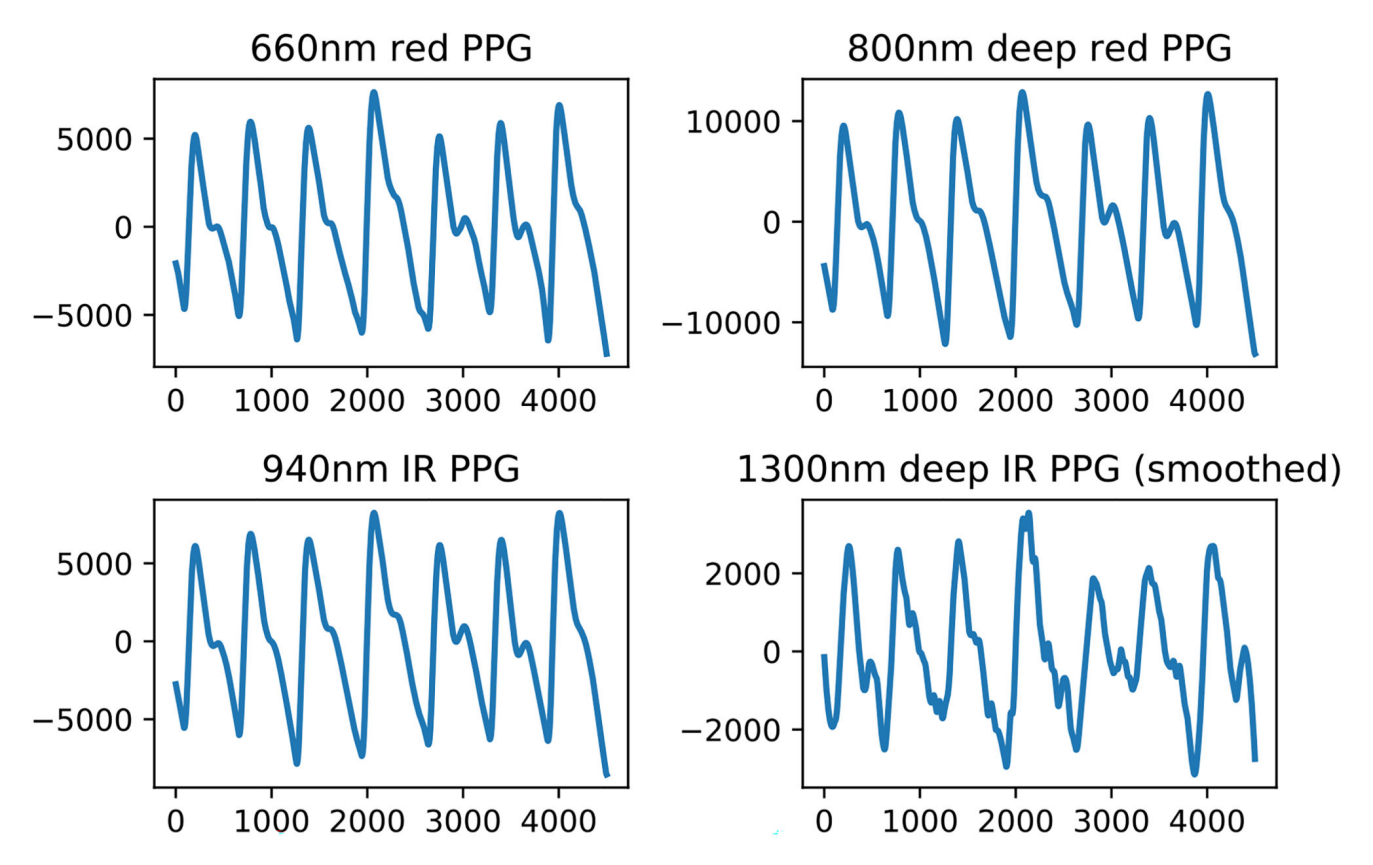
Example filtered waveforms acquired from the finger sensor probe at all 4 wavelengths. For the deep IR wavelength at 1300 nm, water starts to dominate the absorption spectrum, significantly attenuating the transmitted signal and introducing noise. After smoothing the waveform using the Savitzky-Golay filter, the waveform becomes recognisable and both the AC trends are sufficient for extraction of high-level parameters.

The finger sensor probe provided clear PPG at 660 nm, 800 nm and 940 nm after passing through the signal processing pipeline. At 1300 nm, the water becomes the dominant absorbing medium and due to large amounts of water-like fluids surrounding the blood vessels in the human body, the transmitted light signal is heavily attenuated. The resultant waveform is reconstructed using Savitzky-Golay smoothing filter as shown in Fig. 10 together with other fingertip wavelengths, illustrating the difference between them. After reconstruction, the deep IR waveform provides recognisable peaks at the dominant frequency and can be used for ratio-based calculations and peak detection. For algorithms where more precise features like dicrotic notch or area under the curve are required, the clean PPG from other wavelength channels are used.

#### 2) Wrist Recordings

**Fig. 11:**
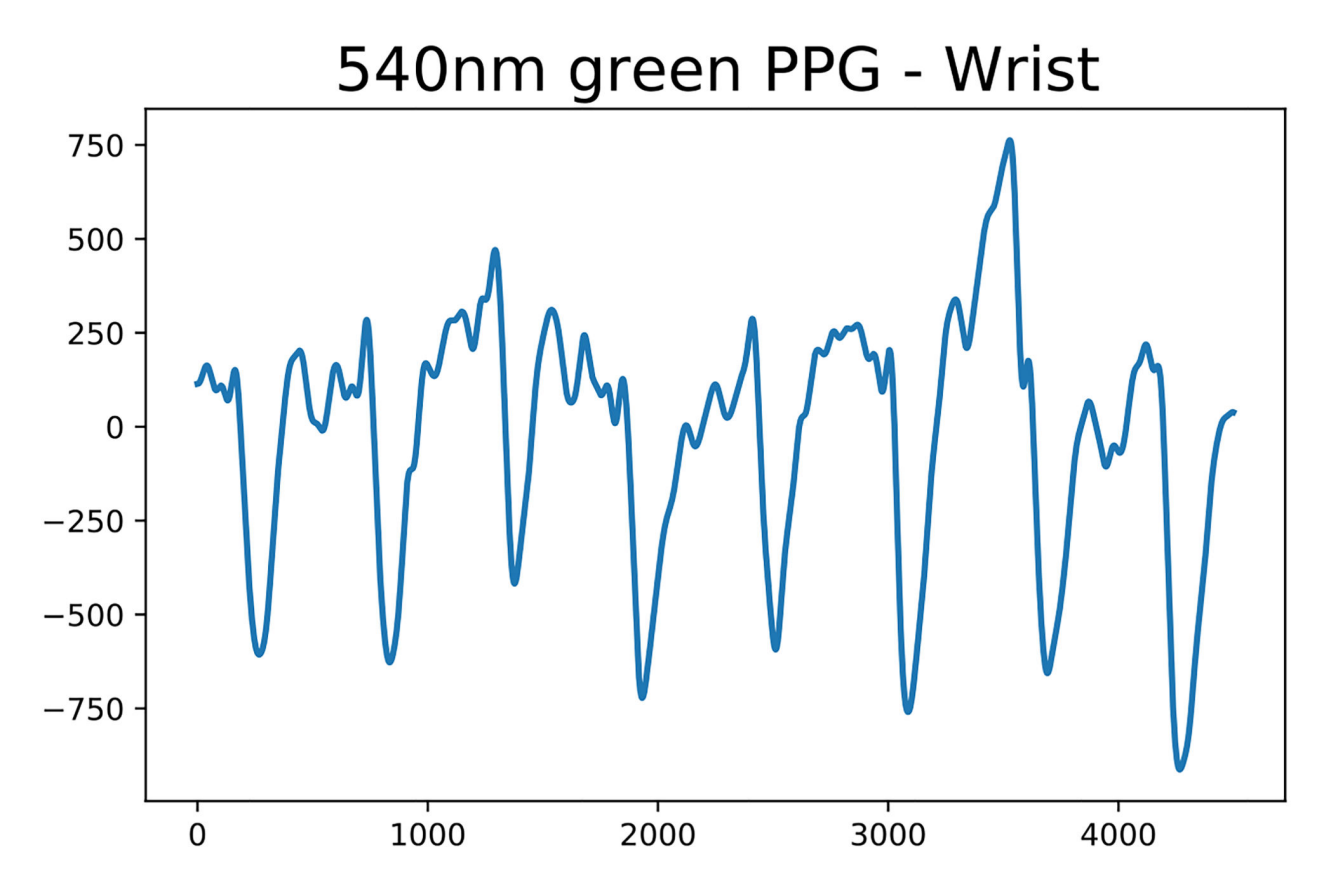
Example waveforms acquired from the wrist sensor probe. The green wavelength has larger amplitude variation thanks to the lower light penetration depth which results in shorter light path throughout tissue and smaller attenuation of the transmitted signal.

The addition of the second sensing location allows the system to extract the time difference between finger and wrist waveform, provided both contains discernible peaks. Due to the increase in noise corruption caused be the reflectance method of waveform acquisition, there is a clear difference in waveform shape between finger and wrist as indicated by literature [26]. We only utilise the green (525 nm) channel as its lower tissue penetration depth makes it less susceptible to noise and in turn more robust for peak detection. The observed waveform in Fig. 11 shows clearly defined peaks corresponding to heart-rate allowing the PWV estimation.

### B SpO_2_ Calibration Experiment

**Fig. 12:**
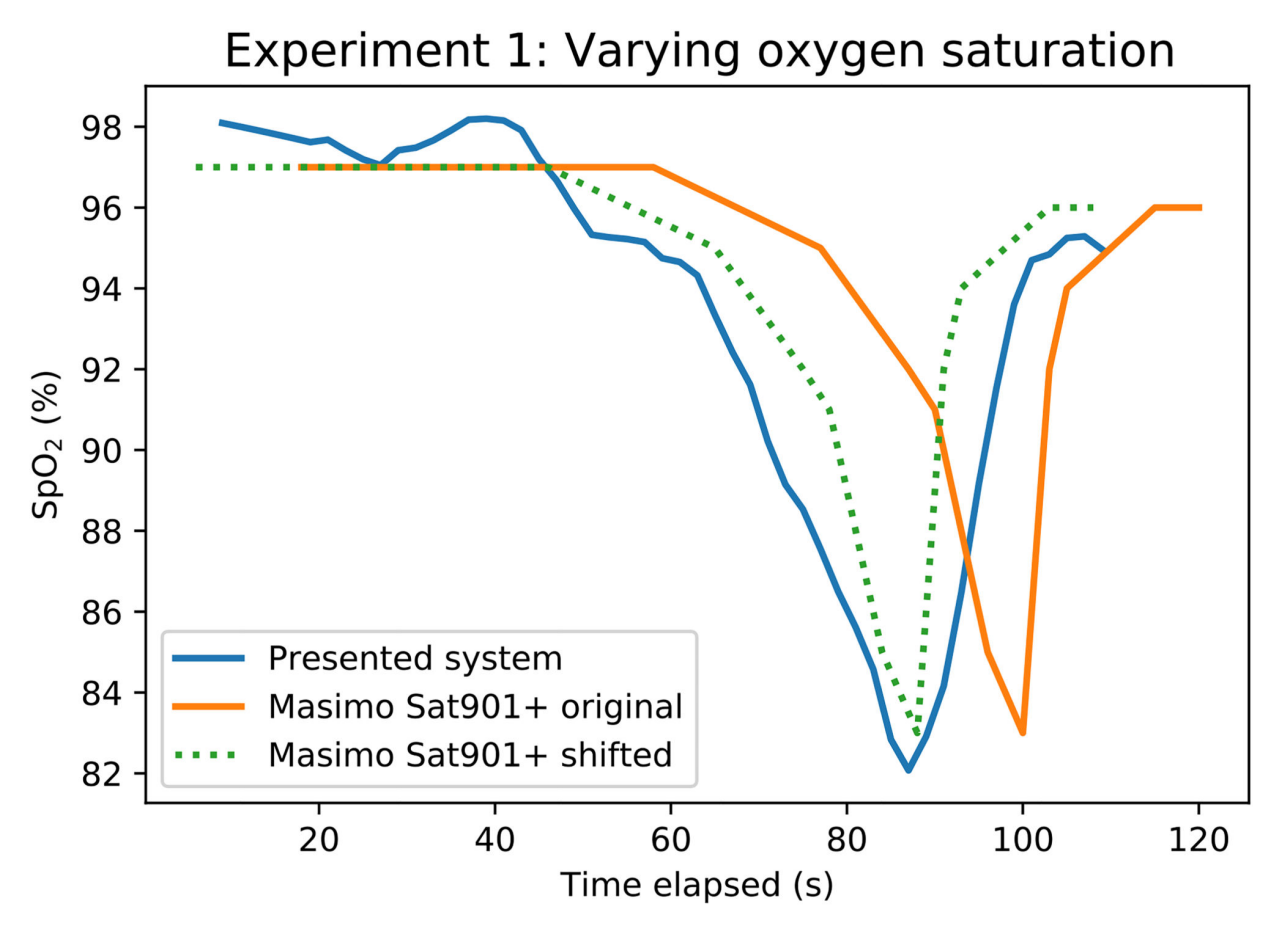
Comparison between the oxygen saturation values obtained from equation (4) using the presented system and displayed values on the Masimo medical pulse oximeter.

The commonly used empirically derived equation relating the red and infrared wavelength ratio and SpO_2_ is defined as [27]:

(4) $${\text{SpO}}_{2}=110-25*R$$

where R has been previously defined in equation (2). A trained experiment subject can momentarily decrease his oxygen saturation in blood by varying their breathing rate, allowing comparison of the accuracy and sensitivity of the proposed system with a medical grade pulse oximeter. Throughout the experiment, continuous values from both sensors were recorded and the resultant SpO_2_ values are plotted in Fig. 12. It is important to note that the Masimo Sat901+ only shows rounded SpO_2_ values and as observed during the experiment, it has a delayed response. The subject was already breathing normally by the time the oxygen saturation started dropping. The observed time delay was around 10 s and it corresponds to the shift shown in Fig. 12. The delay is attributed to the Masimo device software and the use of Masimo’s proprietary algorithms.

In terms of absolute values, once shifted in time, the mean error in SpO_2_ value is 2.51% with a standard deviation of 2.11. The guidelines for performance of medical pulse oximeters by the U.S. Food and Drug Administration (FDA) requires the measured value to be within 4% of the true value [28] obtained invasively. These results show that the measured ratio between PPG waveforms follows the changes in oxygen saturation in blood and therefore the system is capable of measuring pulse oximetry.

### C SpO_2_ and Heart-Rate Measurement

The remaining experiments were performed as part of a clinical study on healthy volunteers at Imperial College London. Each session was 10 minutes long and recorded with participants sitting down and at rest. For PWV calculation, the distance between the two probes was measured for each participant at the start of the session. Every minute throughout the recording, a spot measurement of heart-rate and oxygen saturation was taken from the Masimo pulse oximeter.

To calculate the corresponding HR and SpO_2_ values from acquired raw waveforms, a 5 s segment was isolated at the start of each minute for every subject, leading to 10 datapoints per subject. In some cases, the waveform segment around the 1 minute mark was suffering from motion artefact noise and therefore was omitted.

**Fig. 13:**
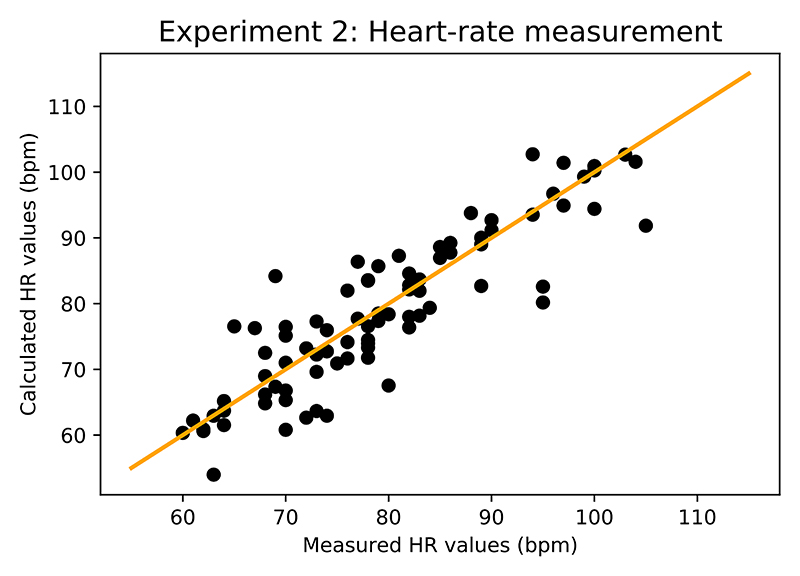
Experimental results for heart-rate monitoring across the subjects. Achieved mean error was 4.08 bpm with standard deviation of 3.72.

Overall, 84 points were analyzed and their comparison to Masimo values is illustrated in Fig. 13. The achieved mean error was 4.08 bpm with a standard deviation of 3.72. It is important to note that the Experiment 1 demonstrated a time lag between values from the Masimo device and real-time continuous values form the presented system. Combined with the variance in subjects heart-rate of up to 20 bpm over the course of a single 10 minute recording, the measured error is within the expectation. The data show that our platform is capable of continuous hear-rate monitoring across the healthy subject cohort.

Obtained SpO_2_ measurement results are illustrated in the Table I. For one subject in particular, the empirical equation (4) does not provide adequate results with close to 5% mean error. However, the low standard deviation indicates that error was mostly caused by a constant offset. The remaining 9 subjects achieved the mean error of 1.54% which resides within the FDA allowed limits [28].

While these results indicate that the empirical equation (4) requires refinement prior to its application across the general population, we have confirmed that our platform produce valid ratio R for the two pulse oximetry wavelengths across the experimental cohort.

### D Analysis of Pulse Wave Velocity (PWV) as a Precursor for Blood Pressure Estimation

**Fig. 14:**
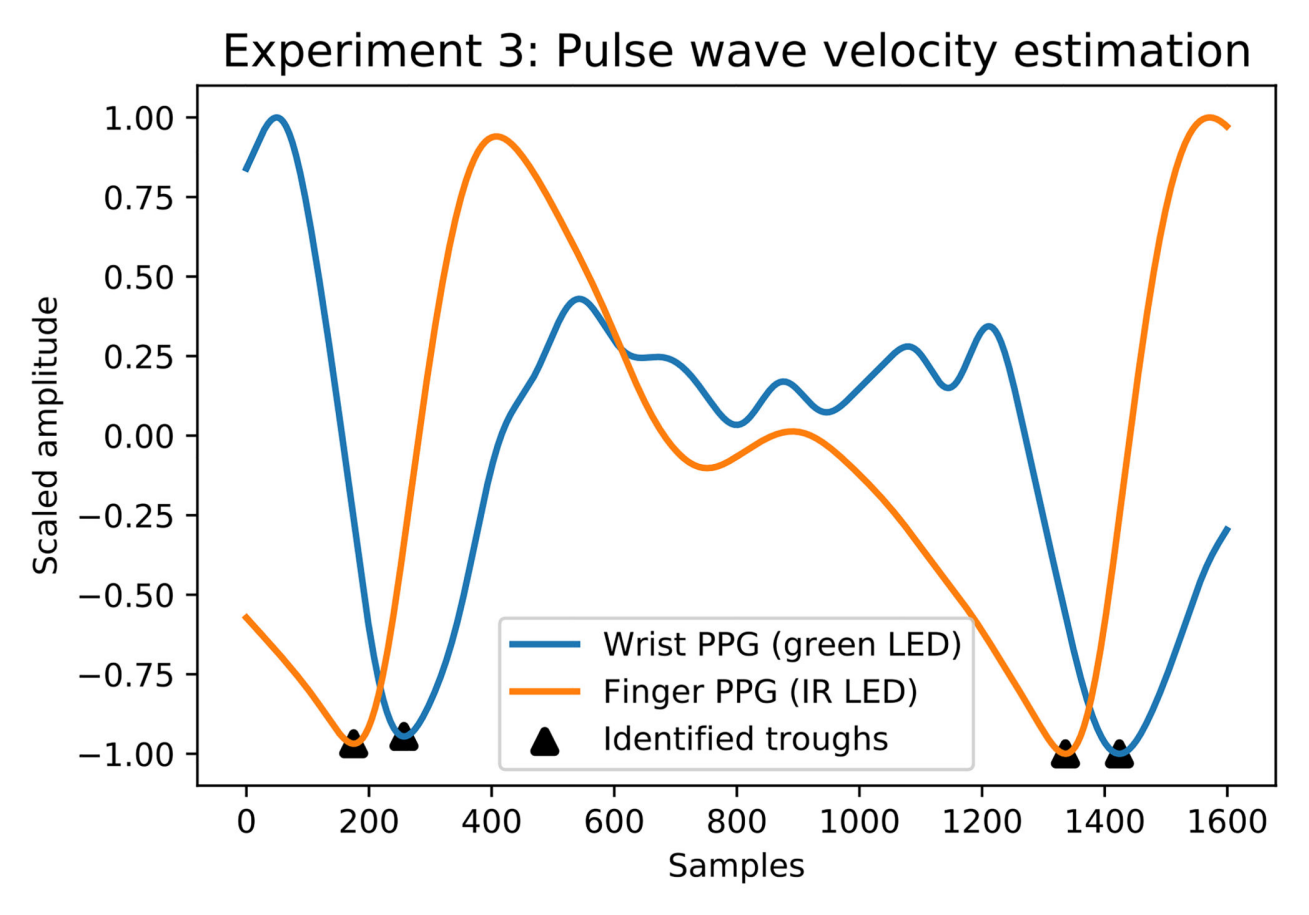
Illustration of time lag between wrist and finger PPG signal using a trough detector.

The Fig. 14 illustrates the approach for extracting the time lag between the wrist and finger waveform. The troughs of both waveforms are identified using a predictive peak detector and the difference between them is computed across the whole recording. A single recording is split into 10 1-minute segments. For a segment to be deemed applicable for PWV extraction, over 10% of the peaks in the same region within both synchronised signals need to be of sufficient quality. Due to the nature of varying shape and noise levels within PPG waveform, and a lack of general DSP tools that can be applied in every situation, the final quality checks were done manually. Sufficient quality for fingertip signal was defined as PPG shape with no deformities including dicrotic notch as illustrated in Fig. 2. For wrist signal, the quality was deemed sufficient in the case where each period was clearly defined by two troughs as shown in Fig. 11. The participant was only scored if at least 50% of segments evenly split across the 10 minute recording were applicable for PWV extraction. Next, we define the relationship between PWV in m/s and trough position difference (*δ*) as:

(5) $$PWV=\frac{d}{\delta +x}\times 1000$$

Where *d* is the finger-wrist distance in meters, *δ* is number of samples between the troughs when sampled at 1 kHz and *x* is constant offset introduced by hardware limitations. Assuming the PWV values within our cohort correspond to the reference values obtained by Diaz et al. [29] we can calculate the constant offset *x* for each participant. We then obtain normalized value *PWV*_*n*_ by subtracting the offset *x* from mean *δ* for each participant and recalculating PWV. The Table II summarizes obtained results.

While all the steps have been taken to reduce synchronization error, the black box implementation of the TI development board and parallel serial port communication introduces a random delay *x* between the two boards every time a new recording is started. It is important to note that this time delay is constant throughout each recording and therefore can be eliminated with initial calibration of the system.

Looking more closely at the results in Table II, we can note significant difference in the calculated value between the first participant and the rest of the cohort. With only 53 individual beats analyzed, this participant was on the edge of acceptability for PWV extraction. Coupled with issues of peak definition even for peaks marked as “good” that introduces variations in the final peak position, we have excluded this participant from the overall summary. The overall weighted average PWV after normalization was 5.80 ± 1.58 m/s matching with the values found for this age group in [29] and showcasing the capability of the system to measure the baseline time difference between the two waveforms.

### E Haematocrit Ratio

**Fig. 15:**
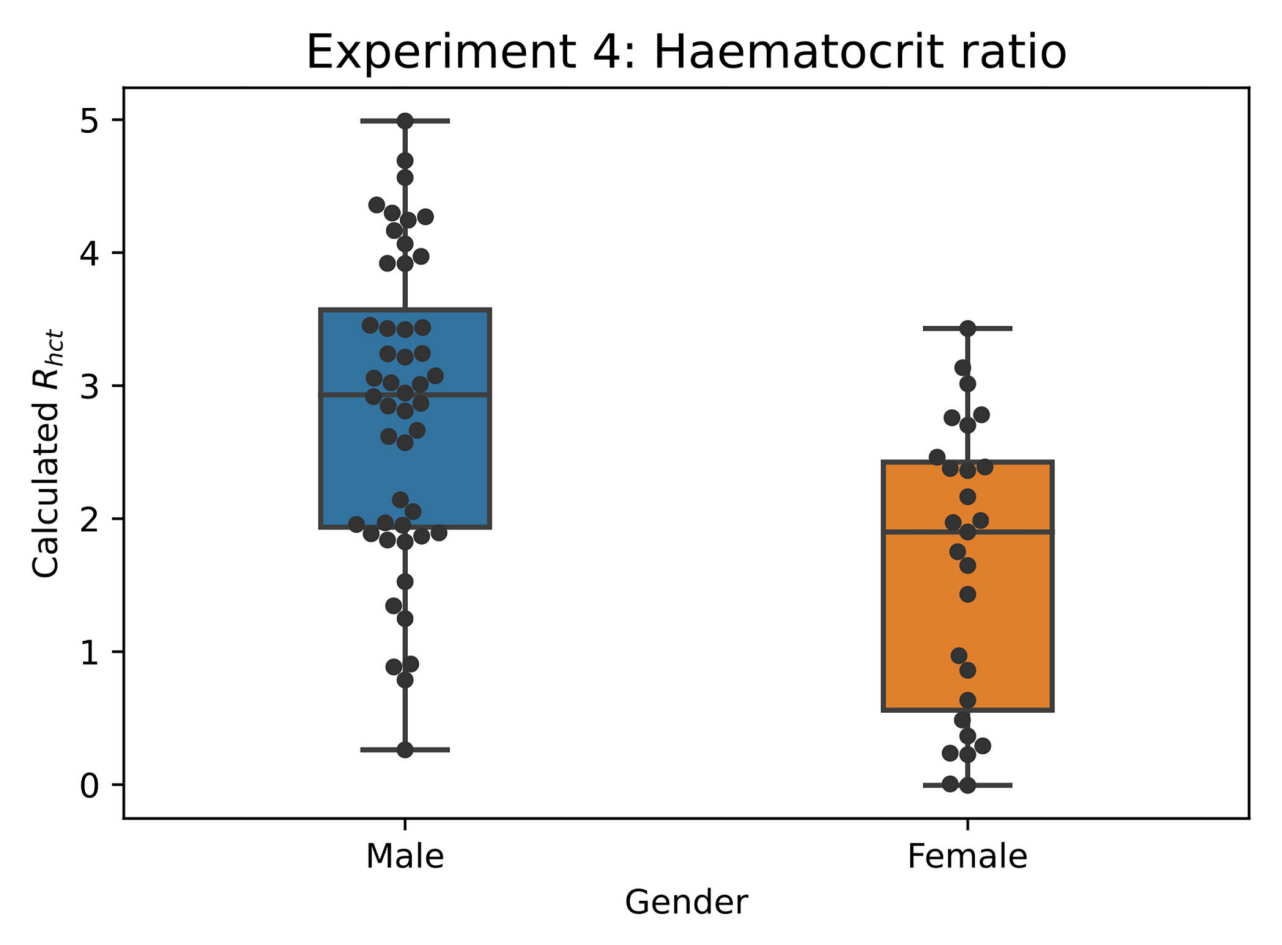
Haematocrit ratio (*R*_*hct*_) plot to illustrate difference between values obtained from male and female participants.

Here we present the raw results when attempting to calculate the Hct ratio without any significant post-processing involved. By calculating the ratio between 800 nm and 1300 nm wave-length as defined by equation 2 we can calculate an average *R*_*hct*_ for each 1 minute segment within the 10 minute recording. Repeating the approach for every participant, removing clear outliers due to noisy data and separating by gender we arrive at the Fig. 15.

Based on the available literature, the reference values for haematocrit are 40–54% for men and 36–48% [30]. We can note that the obtained *R*_*hct*_ values for female participants do overall differ from male values. The median value of *R*_*hct*_ was 2.93 and 1.90 for male and female respectively. The male median value lies outside of the female upper quartile boundary by 0.5 which points to a likely difference between the two distributions. Invasive hct measurements to confirm the calculated values were not part of this experiment and therefore more clinical validation is required before further claims can be made.

## V Device Safety and Comfortability

To better guide the design process and prepare for future clinical studies, each participant was asked to fill a questionnaire for perceived device comfortability at the end of the recording session. The questionnaire consisted of 5 main questions allowing a rating from 0-10 where 10 was the best (agree the most) score. Additional sections for optional written answers were provided as well. The questions were asked as follows:

1) The device today was comfortable to wear for the duration of the study (Scored)
2)
  a) If a smaller (wristwatch-sized) device could monitor my health, I would be happy to wear the device for most of the day (Scored)
  b) What would be the main factors which would influence you wearing the device during the day? (Written)
  c) I would be happy to wear the device when I sleep (Scored)
  d) What would be the main factors which would influence you wearing the device during sleep? (Written)
3) If I was ill, I would wear rather wear a device which can monitor my health safely at home rather than be admitted into a hospital (Scored)
4) I think seeing my own results displayed on the screen would be important. (Scored)
5) We would be grateful for any additional comments – what other features would improve the device? (Written)

**Fig. 16:**
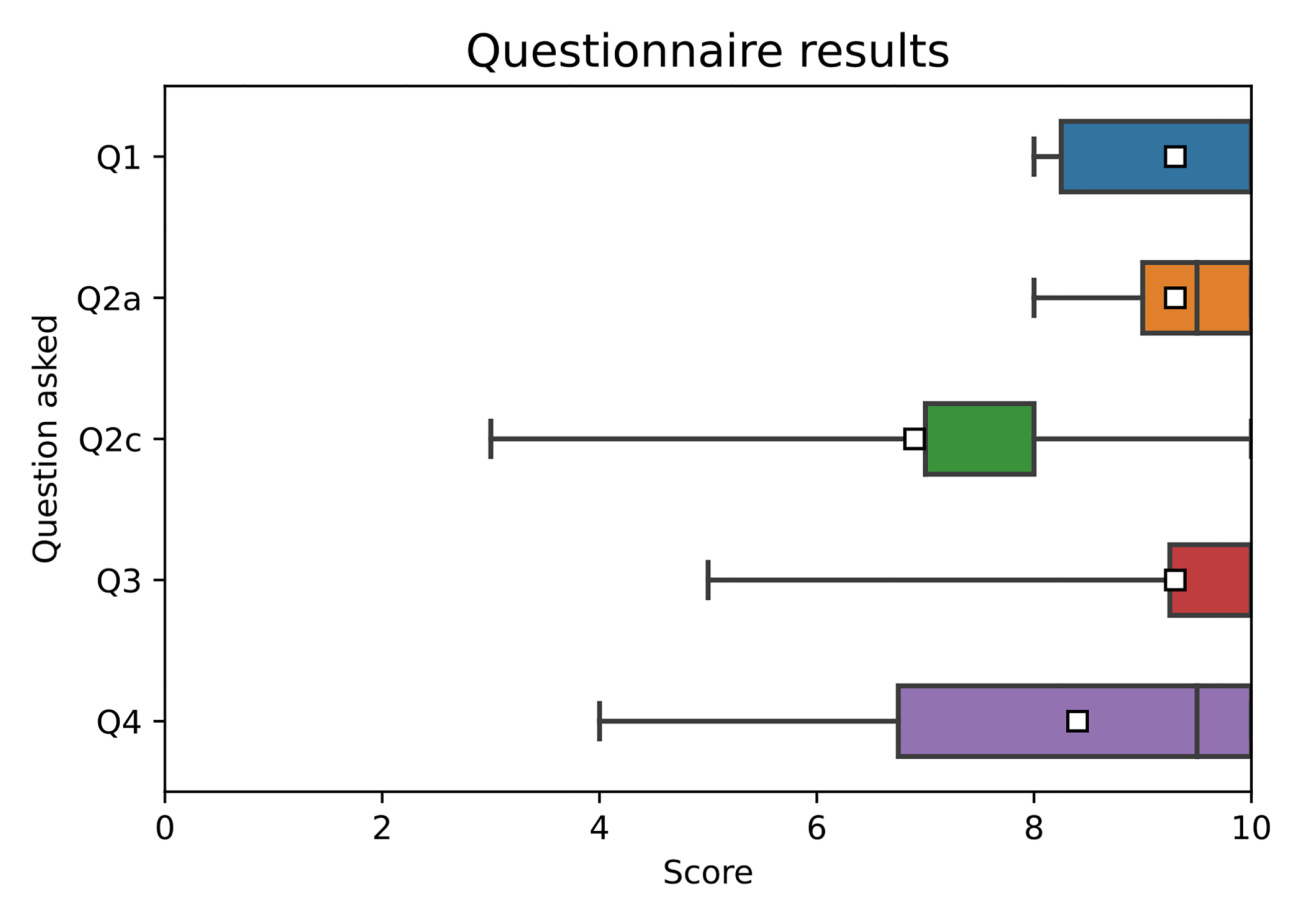
Boxplots of answers to each of the scored questions. The white square signifies mean score value for each question.+.

The aim of the questionnaire was to understand if any changes to the hardware are necessary before it can be used with patients for prolonged periods of time (eg. multiple hours) and how feasible a future wearable implementation would be from the user perspective. The results of the questionnaire are summarized in Fig. 16. The written comments were highlighting the need to improve the comfort level of the fully wearable system as a lot of users were hesitant to use the given prototype overnight as illustrated by the relatively high range of the scoring of question 2c.

The presented system has been reviewed against the IEC60601-1 electrical safety standard to identify any problematic parts before being trialled in hospitals. Labelling and IFU requirements have been satisfied and provided the laptop charger is rated for hospital use, the device has been cleared via a third party review for clinical work.

## IV Discussion

The deliverable of this work can be split into two parts. Number one, we have developed a completely modular PPG acquisition platform allowing continuous, synchronized acquisition from up to 2 different locations with 4 LED wavelength each. The platform comes with a custom software application that controls the system, allows configuration of sensor parameters and saves raw data to file for further analysis. Platform has been prepared for clinical trials in hospital settings by preparing the necessary regulatory paperwork for electrical safety. Number two, we have designed a pair of probes for wrist and finger sensing, specifically aimed at monitoring patients with dengue, but could be applied to other infectious diseases. The wavelength in each probe has been selected such that modalities that correlate with haematocrit, commonly measured in dengue patients as a marker of vascular leak, can be measured. In a series of experiments, we have shown the capability of the system to measure these modalities on a limited number of subjects in lab environment.

Comparing the platform to similar published work, we offer the most flexibility in parameter tuning as well as in richness of information that can be measured. Table III gives an overview of other published examples. Other systems usually focus on measurement of a specific modality [12], [18] and then build the system completely around it. In the presented work, a predefined objective was to allow a combination of all relevant parameters in a single system, allowing applications in multiple diseases. By standardising the sensor probe pin-outs, we open the door for third party partnerships utilising the presented system for research.

It is important to note the limitation of the acquired dataset in this work. The size of the cohort as well as age distribution is heavily skewed towards young, healthy people which is not the same cohort as will eventually benefit from this technology. All data have been collected in a laboratory environment, with subjects at rest, further skewing the outcomes. Motion artefacts were more prevalent in the wrist waveform as hand movement was permitted during the recording to reflect real-life hospital settings. Overall, the data quality was very good, with fingertip PPG having clear heart-rate values for over 90% of all recorded data.

Another important factor is the lack of comparison between the calculated PWV and haematocrit values with ones obtained invasively through cuff-based blood pressure monitor and blood draw. Results shown in both section 4D and 4E while promising, still need more validation against clinical data.

Lastly, we have partnered with Oxford University Clinical Research Unit and the Hospital for Tropical Diseases (HTD) in Ho Chi Minh City, Vietnam to conduct a clinical study on dengue patients and further validate the performance of our system. We will be enrolling mild and severe patients in both children and adult cohorts to obtain representative data for generalised outcomes.

## VII Conclusion

The results prove the platform is capable of heart-rate monitoring, oxygen saturation monitoring and provides time delay between finger and wrist waveform as well as adequate deep infra-red PPG waveform at 1300 nm. When compared with the Masimo medical device, we have achieved the mean error of 4.08 ± 3.72 bpm for heart-rate and 1.54 ± 1.04 for SpO_2_. Taking into consideration the synchronisation offset between the Masimo device and our real-time data, these results showcase that the presented system sensing capabilities are on par with those of a medical device. Furthermore the PWV experiment showed that the system was capable of recording the time offset between 2 synchronized probes despite the hardware imperfections introducing an additional delay. The calculated *R*_*hct*_ that serves as a surrogate for haematocrit estimation has shown significant difference between male and female values as supported by relevant literature on hct values in healthy population. The supplied questionnaire proved that presented device was comfortable to be wear and gave insights into the next design steps. In conclusion, we presented and validated the first PPG-based system utilising 8 independently configurable channels that demonstrate sensitivity to HR, SpO_2_, PWV and haematocrit.

The system was built using components selected to allow implementation of the system into a fully wearable plat-form. Low-cost and off-the-shelf availability of the components enables device mass-production and deployment for large-scale patient management in low-resource settings. With the recent shifts in medicine to remote patient monitoring and individually-tailored treatment, our wearable platform will be able provide richer data than conventional PPG systems to allow accurate and real-time monitoring of patients especially during outbreak and peak seasonal epidemics of dengue and other infections.

## Figures and Tables

**Fig. 1:**
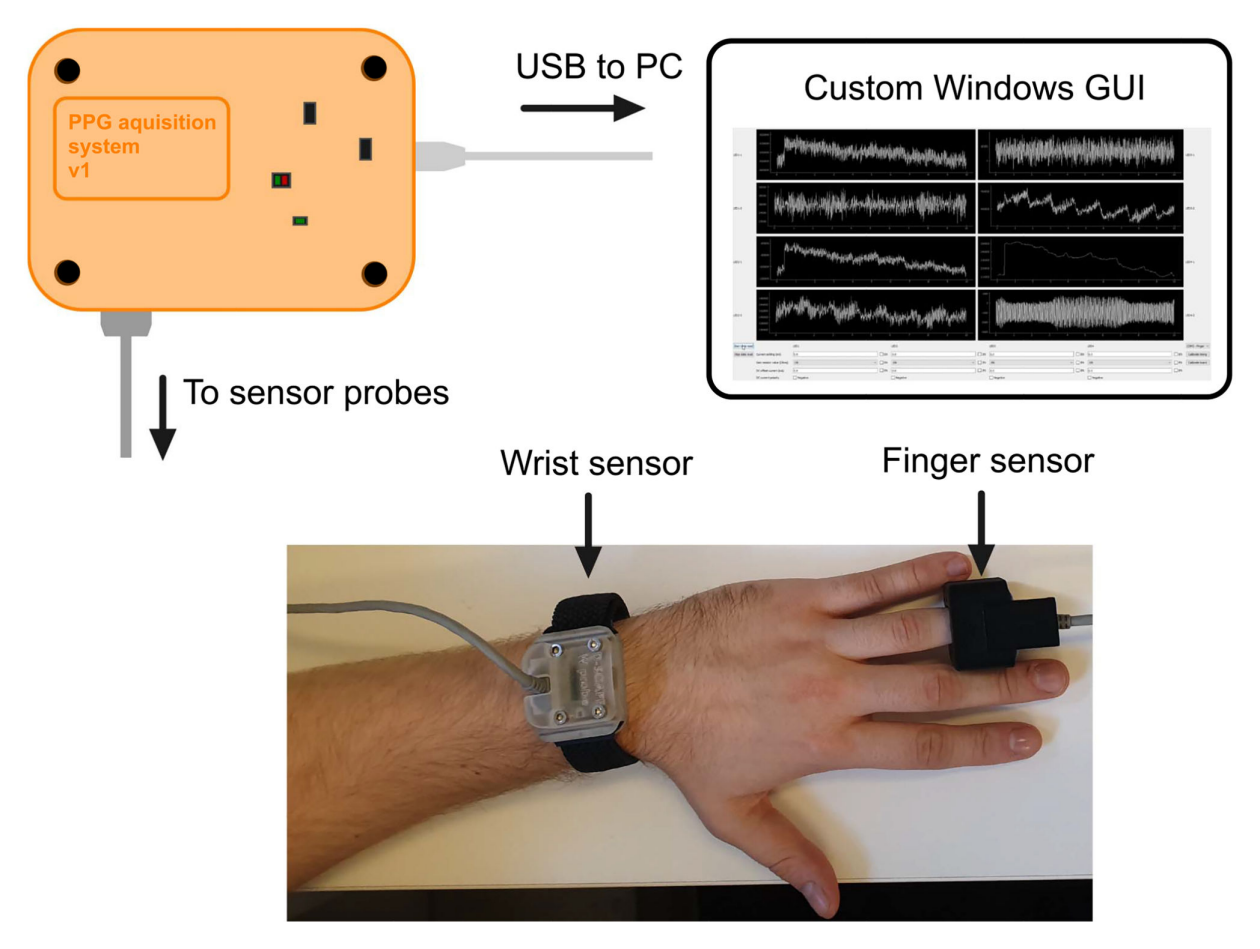
Illustration of the presented PPG acquisition platform.

**Table I:** Table of S_P_O_2_ Measurement Results in Healthy Volunteers

	Across all (%)	Excluding 10th subject (%)	10th subject only (%)
**Mean error**	1.85	1.54	4.86
**Standard deviation**	1.41	1.04	0.82

**Table II:** Summary of PWV Experiment Where *PWV*_*n*_ is the Normalized PWV Value After Removing Offset *x, σPWV*_*n*_ is a Standard Deviation of *PWV*_*n*_, *d* is Finger-Wrist Distance and *x* is the Random Offset Introduced by Hardware When Starting the Recording

ID	Finger-wrist distance (cm)	$PWV_{n}\pm \sigma_{PWV_{n}}(\text{m/s})$	*x* offset (samples)	No. of beats analyzed
1	17.1	7.31 ± 3.60	87.90	53
2	15.9	5.45 ± 0.81	8.17	131
3	21.0	5.53 ± 1.32	121.49	138
*4*	20.5	5.52 ± 0.84	21.61	80
5	20.7	6.66 ± 2.09	16.65	76
6	18.5	6.54 ± 2.24	45.74	141
7	18.1	Wrist data too noisy	N/A	N/A
8	16.9	Wrist data too noisy	N/A	N/A
9	20.9	5.44 ± 1.19	169.17	180
10	20.0	Wrist data too noisy	N/A	N/A

**Table III:** Comparison Against Other Published PPG Systems

Identifier	Separate PPG channels	Light wavelengths	Sampling rate	Het estimation	Measurement locations	Ref
**1**	1	1 (940nm)	200 Hz	No	Wrist	[31]
**2**	2	1 (660nm)	20,000 Hz	No	Finger and wrist	[12]
**3**	5	5 (670nm - 1300nm)	120 Hz	Yes	Finger	[18]
**4**	8	5 (530nm - 1300nm)	1,000 Hz	Yes	Finger and wrist	**This work**
